# Bridge Over an Aging Population: Examining Longitudinal Relations Among Human Resource Management, Social Support, and Employee Outcomes Among Bridge Workers

**DOI:** 10.3389/fpsyg.2018.00574

**Published:** 2018-04-26

**Authors:** Klaske N. Veth, Beatrice I. J. M. Van der Heijden, Hubert P. L. M. Korzilius, Annet H. De Lange, Ben J. M. Emans

**Affiliations:** ^1^HRM, Professorship Sustainable HRM, Hanze University of Applied Sciences, Groningen, Netherlands; ^2^Institute for Management Research, Radboud University Nijmegen, Nijmegen, Netherlands; ^3^Open University of the Netherlands, Heerlen, Netherlands; ^4^Kingston Business School, Kingston University, London, United Kingdom; ^5^Faculty of Psychology, NTNU, Trondheim, Norway; ^6^Hotel School of Management, University of Stavanger, Stavanger, Norway; ^7^Human Resource Management, HAN University of Applied Sciences, Nijmegen, Netherlands; ^8^Institute of Business Administration and Technology, University of Groningen, Groningen, Netherlands

**Keywords:** bridge employment, HRM, social support, employee outcomes, leader-member exchange, work engagement

## Abstract

This two-wave complete panel study aims to examine human resource management (HRM) bundles of practices in relation to social support [i.e., leader–member exchange (LMX), coworker exchange (CWX)] and employee outcomes (i.e., work engagement, employability, and health), within a context of workers aged 65+. Based upon the social exchange theory and the Job Demands-Resources (JD-R) framework, it was hypothesized that HRM bundles at Time 1 would increase bridge workers' outcomes at Time 2, and that this relationship would be mediated by perceptions of LMX and CWX at Time 2. Using a longitudinal design, hypotheses were tested in a unique sample of Dutch bridge employees (*N* = 228). Results of several structural equation modeling analyses revealed no significant associations between HRM bundles, and social support, moreover, no significant associations were found in relation to employee outcomes. However, the results of the best-fitting final model revealed the importance of the impact of social support on employee (65+) outcomes over time.

## Introduction

Due to the graying and dejuvenization of the global workforce (Bal et al., 2015; Müller et al., 2015), older workers are stimulated to continue to work. For example, in the Netherlands until 2013 the official retirement age was 65 years, and is ever since gradually increased by the government to age 66 by 2018 and 67 years by 2021. Financial stipends are given to employers to stimulate work for 65+ workers, making the so-called bridge employment for employers as well as older workers financially interesting. However, in recent studies late-career issues have been largely ignored as opposed to mid-career ones that have increasingly been examined (e.g., Veth et al., 2015). The proliferation of concepts such as the protean career (e.g., Hall, 2004) and the sustainable career (Van der Heijden and De Vos, 2015), and the life-span development model of careers (Savickas, 1997; Baruch, 2004) has made each career stage a viable area of inquiry, including late careers. An employee's mid-career stage is neither no longer simply seen as a time of maintenance, nor is one's late-career stage just seen as a time of decline and obsolescence, herewith pushing somebody toward retirement. Instead, Shultz and Wang (2008) denoted “a much greater appreciation for the continued potential for growth and renewal of workers in their mid to late careers” (p. 113). Therefore, in this empirical study, we endeavor to shed light on the challenges the growing amount of workers in the late stage of career, the so-called bridge workers, have to cope with. Following Shultz (2003), we state that bridge employment refers to the labor force participation patterns exhibited by older workers that characterize the transition from late careers jobs toward complete labor force withdrawal. Bridge work can include part-time and fulltime work, seasonal work, but also temporary work (Wang et al., 2009).

Based on previous empirical research (e.g., Huselid et al., 1997; Becker and Huselid, 1998; Wright et al., 2001), it has become clear that human resources management (HRM) can contribute to a firm's effectiveness and competitive advantage. It is assumed that particularly bundles of HRM practices, rather than individual HRM practices, can be sources of sustained competitive advantage, because these bundles are often unique, and difficult to imitate (Lado and Wilson, 1994; Ferris et al., 1998). Earlier scholarly work on the so-called HRM-organizational performance linkage showed a fully mediating role of employee outcomes (Zhang and Morris, 2014). Employee outcomes are the most immediate consequence of HRM, while organizational performances are more distal to HRM and are less directly influenced (Purcell and Kinnie, 2007; Wright and Nishii, 2007; Guest and Conway, 2011). More empirical research is needed in order to enhance our understanding of the role of HRM in the light of bridge workers in particular. This study examines longitudinally individual employee outcomes, such as work engagement, caused by organizational-level HRM practices, like flexible worktime and permanent development in one's function. In this regard, HRM can be viewed as a subsystem that exchanges information and energy with the environment and that is aimed to attract, develop, motivate, and retain employees who are able to ensure the effective functioning and survival of the organization (Jackson and Schuler, 1995; p. 238). As such, our approach is in line with social context theory, which states that HRM policies and practices are influenced by relevant social, economic, cultural, and political context (Ferris et al., 1998). More specifically, in the 1990s organizations endeavored increased competition by relocating mass production worldwide or by outsourcing peripheral work (Pennings and Sleuwaegen, 2000). However, most research focusing on bridge employment as a beneficial opportunity for organizations, loomed after 2000, when the retirement of Baby Boomers began to generate workforce shortages (e.g., AARP, 2005). In the current vulnerable economic situation, we expect a higher prevalence of HRM that is characterized by maintenance practices as an awaiting-for-retirement-approach and that is focused on retaining the older worker on his/her current level of functioning. In addition, a higher amount of retirees may be hired as contingent workers because organizations will need to maintain flexible access to a skilled and experienced workforce (Greller and Stroh, 2003).

Over the past decades, research on HRM has made considerable advancement in understanding linkages between certain HRM practices and employee outcomes, such as work engagement, employability, and perceived health (e.g., Clarke and Hill, 2012; Samnani et al., 2012; Alfes et al., 2013). It goes without saying, that a further focus on the question of how employees perceive the HRM practices provided, rather than only relying on intended HRM practices is desirable (e.g., Khilji and Wang, 2006; Nishii et al., 2008). Earlier, it has been shown that measures of perceived and actually used HRM practices differ substantially from intended, implemented HRM practices (Gratton and Truss, 2003; Conway and Monks, 2008; Snape and Redman, 2010; Wright and McMahan, 2011). Hence, perceived availability and actual use of HRM bundles will be examined in this new survey study.

Although empirical findings have generally confirmed the existence of a significant relationship between perceived availability and actually used HRM on the one hand, and positive individual and organizational outcomes (e.g., Paauwe, 2009) on the other, there have been repeated calls to transfer the prime focal point to an investigation of factors that may intervene in the relationship between HRM and individual and organizational outcomes (Ferris et al., 1998; Ostroff and Bowen, 2000; Wright et al., 2003; Boselie et al., 2005). Hence, more insight into how (i.e., through which mediators) HRM may impact these outcomes (the so-called “black box,” Becker and Gerhart, 1996; Guest, 1997; Ramsay et al., 2000) is therefore considered as one of the key issues in the HRM research field. Despite the efforts in earlier research (e.g., Kuvaas, 2008; Snape and Redman, 2010) to solve the “black box” by examining the mechanisms through which HRM practices impact upon employees outcomes, more research is still required, particularly on the aforementioned relations among 65+ employees (Alfes et al., 2013).

In line with the social exchange theory (Gouldner, 1960; Blau, 1964), we argue that by providing HRM bundles the line managers in organizations send obvious and implicit signals to their employees on how they are valued and trusted. In turn, these perceived and used HRM bundles may cause feelings of obligation on the part of employees, who might then reciprocate through high levels of performance (Coyle-Shapiro et al., 2004; Gould-Williams, 2007; Purcell and Hutchinson, 2007). In addition, we draw on the job demands-resources (JD-R) model (Demerouti et al., 2001; Bakker et al., 2005) to identify resources that are critical for positive employee outcomes, and the HRM bundles that build and support those resources (see also Snell et al., 1996; Wright et al., 2001). We therefore consider HRM bundles provided at the organizational level—whether perceived or actually used—to be prerequisites of work-related aspects at the individual level (i.e., resources, such as support from one's direct supervisor), which in turn, impact employee (65+) outcomes (i.e., work engagement). So, HRM practices offered at the relatively more distant organizational level that are consequently perceived in a certain way and actually used by individual employees, will be associated with higher employee outcomes. This relationship might be mediated by the more closely related employee's working environment. Theoretically, HRM practices provide employees with the tools or practices that they need to foster their job resources, and ultimately employee outcomes, such as work engagement (Bakker et al., 2005). Up until now, most of this research was conducted using cross-sectional designs. However, it is impossible to establish causality in such designs and cross-sectional studies are not equipped to exclude reversed causal relationships (Zapf et al., 1996). In particular, the longitudinal study that is reported on in this contribution was used to determine changes that HRM practices entail in employee outcomes through social support being the mediator, and by excluding time-invariant and unobserved third variable influences. Our objective to include changes over time asked for a longitudinal study design in order to be able to capture intra-individual age effects.

This study endeavors to overcome the aforementioned limitations of previous research by contributing to our understanding of how employees' perceptions and actual use of HRM practices are linked with employee outcomes by proposing social support as an underlying mechanism in the workplace that mediates the aforementioned relations. The first objective of this two-wave panel study is to examine the direct longitudinal effects of perceived and actually used HRM practices on employee outcomes over time. Secondly, we will test the relations between HRM and employee outcomes (Evans and Davis, 2005; Wright and Nishii, 2007) and examine potential mediating effects of social support between HRM and employee outcomes, among a sample of employees aged 65 years and older. Before addressing the specific hypotheses of this study, we will pay attention to our main concepts to be examined: perceived and actually used human resource management practices, employee outcomes, and to the mediating role of social support of supervisors and coworkers.

## The impact of HRM on employee (65+) outcomes

Prior research has shown that HRM practices can influence attitudinal and behavioral outcomes at the individual level (Guest, 1999; Gerhart et al., 2000; Gratton and Truss, 2003). Furthermore, perceived and actually used HRM practices may be more proximal predictors of individual attitudes and behaviors than intended HRM practices as reported by managers (e.g., Khilji and Wang, 2006; Nishii et al., 2008; Kooij et al., 2010). Hence, it may be more meaningful to focus on HRM practices that are actually perceived as available and/or used by employees. Following this line of reasoning, this study incorporates both actually used as well as employees' perceptions of HRM practices.

Furthermore, when looking at the mutual employee and organizational relationships, research has examined HRM practices at the individual level, such as regular training and development programs (e.g., Boselie et al., 2005; Kooij et al., 2010; Veth et al., 2017). However, since employees are typically exposed to a range of HRM practices simultaneously (e.g., Wright and Boswell, 2002), it has been argued that these need to be considered holistically in bundles instead of individual practices (Gould-Williams and Mohamed, 2010; Snape and Redman, 2010). That is to say, as individual HRM practices can conflict, nullify, substitute, or complement each other, we will focus on examining effects of combinations of HRM practices, so-called HRM bundles, on employee outcomes.

Our choice of bundles of HRM practices is in line with the conceptually meaningful distinction between maintenance and development bundles of HRM practices made by Kooij et al. (2010). These bundles of HRM practices have been classified according to the shared goals of the specific HRM practices they entail. Based on earlier research (Higgins, 1997; Baltes et al., 1999; Carstensen, 2006) it was shown that throughout the life-span people allocate different resources to reach their goals. According to life-span theory of motivation, the socioemotional selectivity theory (SST; Carstensen et al., 1999) humans adjust time horizons with increasing age. Hence, the authors revealed that although chronological age can function as an index of time, a second index becomes salient as people grow older, namely the subjective sense of remaining time until death. Goal-directed behavior relies inherently on perceived future time, which is inextricably related to goal selection and goal pursuit. The Regulatory fit theory (Higgins, 1997, 2005) suggests that a fit between orientation to a goal and the means used to approach that goal, produces a state of regulatory fit which both creates a feeling of rightness about people's goal pursuit. People who experience time as rather limited, show a prevention focus and base their orientation to a goal on safety and responsibilities. In contrast, people who experience time as open-ended, show a promotion focus and concentrate on accomplishments and gains (Lang and Carstensen, 2002). Therefore, we can state that these life-span goals can be “translated” (Kooij et al., 2010; p. 1115) into goal orientations with a more promotion or more prevention focus (Higgins, 1997). Thus, maintenance HRM practices are conceptualized as practices focused on retaining employees at their current levels of functioning, or, practices that are rather focused on returning to previous levels of functioning after a loss. The latter being related to protection, prevention, and safety. In contrast, development HRM practices are conceptualized as practices focused on achieving higher levels of functioning, and are related to growth, advancement, and accomplishment (Kooij et al., 2010). Since this study has been conducted among bridge workers, one may hypothesize that, due to the “healthy worker effect,” economically active 65+ workers use both kind of HRM bundles. A review of the aging workforce literature (e.g., Goldberg, 2005; Morton et al., 2005; Hedge et al., 2006) identified seven HRM strategies targeting older employees, such as flexible working options, training and development opportunities, performance evaluation, compensation, and pre-retirement options. The major aim of this review was to identify the importance of different HRM practices for 65+ workers. In this study, we have chosen to incorporate bundles of practices consistent with this maintenance-development approach and cross-validate bundles of HRM practices among a sample of 65+ workers.

The social exchange theory (Blau, 1964) can be used to clarify the relationship between perceived availability and use of HRM bundles and employee outcomes, such as work engagement, employability, and health. The social exchange theory, comprising the norm of reciprocity (Gouldner, 1960; Cropanzano and Mitchell, 2005), suggests that one party in the exchange relationship will reciprocate positively to the other party, and in doing so, this will improve the quality of the relationship. Thus, social exchange theory provides a solid theoretical basis to imply that organization's investments in terms of HRM opportunities that are consequently perceived or actually used by employees, will be reciprocated by employees in terms of positive attitudes and behaviors, for instance being more engaged (Sun et al., 2007; Shaw et al., 2009; Shore et al., 2009). In addition, the Job Demands-Resources (JD-R) framework (Demerouti et al., 2001; Bakker et al., 2005) stated that job resources play a vital role in the development of engagement (see Bakker and Demerouti, 2008; De Lange et al., 2008). For instance, job resources like social support play a crucial role in promoting positive work outcomes, such as work engagement. As defined by Demerouti et al. (2001 p. 501) job resources refer to those physical, psychological, social, or organizational aspects of the job that: reduce job demands and the associated physiological and psychological costs; are functional in achieving work goals; or stimulate personal growth, learning, and development (e.g., autonomy or social support at work). Job resources can either increase employees' growth, learning and development, or help them in achieving work goals (e.g., Schaufeli et al., 2009).

Several studies have demonstrated empirical evidence that successful goal accomplishment is related to *work engagement* (Kahn, 1990; Llorens et al., 2007; Bakker, 2009). Engaged employees, characterized by having a positive and fulfilling state of mind (Bakker and Demerouti, 2007; Schaufeli and Salanova, 2007), have obtained high levels of absorption, vigor and dedication. Absorption refers to being fully immersed and happily involved in one's work. Vigor is characterized by high levels of energy and mental resilience while working. Dedication refers to a sense of one's significance, enthusiasm, inspiration, pride and challenge at work. For instance, a study by Salanova et al. (2005) exhibited the positive impact of work engagement. They concluded that work engagement predicted service climate, which, in turn, predicted employee performance and, subsequently, customer loyalty among Spanish hospitality employees.

In addition, due to the ever-increasing demands of present work life, *employability* is also emphasized. Employability comprehends “the ability to obtain a job and to keep employment, within or without one's organization, for one's present or new customer(s), and with regard to future prospects” (Van der Heijden et al., 2009; p. 156). A huge amount of expertise within (a) certain occupational field(s) forms the keys to guaranteeing one's employability (Thijssen et al., 2008) as it enables employees to cope with fast changing job requirements (Van der Heijde and Van der Heijden, 2006; Van der Heijden et al., 2009). Though specific employability approaches differ according to the perspective that is taken (i.e., society, company, or individual worker) employability is generally referred to as a positive outcome (Thijssen et al., 2008) which appears to be advantageous for both organizational and employee outcomes (Fugate et al., 2004; Van Dam, 2004; Rothwell and Arnold, 2007).

The World Health Organization. (1948, p. 28), defined *health* as: “a state of complete physical, mental, and social well-being and not merely the absence of disease or infirmity.” In an increasingly aging world, we see a growing need for effective, affordable health promotion (Pesek et al., 2010). In line with Schaufeli et al. (2008) who found that engaged employees generally enjoy good mental health, and following (Parent-Thirion et al., 2007), who related the demanding working environment negatively to health and job performance, good health is closely related to positive work affect (World Health Organization., 1948; Demerouti et al., 2001; Rothbard, 2001; Bakker and Demerouti, 2008; Strijk et al., 2009). We therefore argue that employees perceiving and using high levels of HRM, show more positive employee outcomes, and hypothesize the following:

**Hypothesis 1:** Perceived availability and actually used maintenance and development HRM bundles are positively related to employee outcomes over time in a sample of bridge workers.

## The mediating influence of social support in the associations between HRM and employee outcomes

Building upon the social exchange theory (Gouldner, 1960; Blau, 1964) and the JD-R framework (Demerouti et al., 2001; Bakker et al., 2005), we assume that the associations between HRM and employee outcomes may vary by social support (i.e., from one's direct supervisor and one's near colleagues). First, a supervisor has an exclusive relationship with every employee (Graen and Uhl-Bien, 1995), which is developed over time as a result of role expectations and mutual agreements between leaders and members (Volmer et al., 2012). A high-quality relationship, specified by positive reciprocal exchanges between leader and member (Blau, 1964; Kelley and Thibaut, 1978), is connected to various positive outcomes, such as better accomplishment, more commitment, job satisfaction, and a higher degree of mutual liking (Liden et al., 1997; see Gerstner and Day, 1997; Ilies et al., 2007). Therefore, one's immediate manager (team leader or supervisor) can significantly influence a subordinate's attitudes and behavior (Liden et al., 1993; Gerstner and Day, 1997; Ilies et al., 2007). So-called leader-member exchange (LMX) research has been among the most fruitful areas in the leadership literature for decades (Graen and Uhl-Bien, 1995; Schriesheim et al., 1999; Ilies et al., 2007), and is defined in terms of how leaders develop tacit exchange agreements with their members (Graen and Scandura, 1987); the stronger the LMX relationship, the better the worker's relationship is with his or her manager or supervisor. Thus, LMX is a relational approach to leadership and captures the quality of interactions between a supervisor/leader and a supervisee/follower (Graen and Uhl-Bien, 1995; Nishii and Mayer, 2009). Hence, LMX is characterized as one type of exchange that is part of a larger network of exchanges, including coworker exchanges (CWX).

Second, social support, not only from an individual's immediate supervisor, but also from his or her colleagues, is an important mechanism through which workers can reinforce the impact of the perceptions and actual use of HRM bundles, which can lead to increased perceptions of sustainable work outcomes. CWX refers to the quality of relationships between an employee and coworkers (Sherony and Green, 2002), and comprises a dyadic process that is operationalized along similar dimensions as LMX. Whether employees are involved in a high- or low-quality relationship with their supervisor influences their perceptions of their standing within the group (Cogliser and Schriesheim, 2000; Liden et al., 2006; Nishii and Mayer, 2009). Evidence has suggested that when an employee is involved in a high-quality LMX relationship, this influences how other employees accept this individual. If the leader has accepted someone, others will be more likely to accept this person as well (Schyns et al., 2005; Nishii and Mayer, 2009) and allow them to be a part of the so-called in-group (Murphy and Ensher, 1999; Nishii and Mayer, 2009). Therefore, beliefs about respect, trust, and loyalty within CWX relationships are likely to be the same as those same issues in LMX.

**Figure 1:**
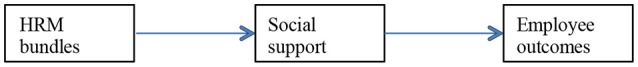
Conceptual model.

We aimed to contribute to the examination of possible pathways through which HRM bundles are related to work outcomes that enhance employees' outcomes, such as work engagement. Therefore, we will examine the mediating role of social support in the relationship between perceived availability and used HRM on the one hand, and employee outcomes on the other hand over time (see Figure 1 for the specific research model). We hypothesized the following:

**Hypothesis 2:** Social support mediates the cross-lagged relationship between perceived and used HRM and employee outcomes over time in a sample of bridge workers.

## Methods

### Sample and procedure

Online surveys were initially sent to all registered clients of a temporary agency specialized in contracting workers of 65+ (*N* = 6,538 working and non-active clients; 74.80% males, mean age = 69.70 years). In May 2011, 784 Dutch workers responded to an online questionnaire, which served as Wave 1 of the study (response rate was 11.99%). For Wave 2 (May 2012), all registered contractors of the agency were invited to participate again (*N* = 6,538 working and non-active clients). Of these individuals, 655 completed the online questionnaire at Time 2 (response rate was 10.01%). All respondents with missing data were eliminated, resulting in a final dataset of 228 participants, who filled out both the Time 1 and Time 2 measurement.

Concerning the demographics from Wave 1, 76.50 percent of the respondents were male, and their mean age was 69.20 years (*SD* = 6.54 years; range 60–85 years). Of the participants 91.2% were older than 65 years. On average, the respondents worked 2.90 (*SD* = 3.53) years for the employment agency, while these participants had worked on average 34.18 (*SD* = 16.07) years prior to their 65th birthday. Participants worked on average *M* = 14.25 h per week (*SD* = 15.20) for the temporary employment agency. The majority had a bridge employment position in the education & science sector (27.6%), followed by transportation & delivery (18.2%), and technology (10.5%). Importantly, comparative analyses of the respondent and total group of employees revealed that the sample did not differ significantly in terms of age and gender from the total employee population working for the temporary employment agency (see also Müller et al., 2013; Baltes et al., 2014). To reduce possible common-method bias, we used scales with different scale ranges and answering categories. In addition, we controlled for common method bias statistically by using a factor analysis without rotation that included all 67 Likert items (Podsakoff et al., 2003). Administration of Harman's single-factor test revealed 11 factors that explained 70.9% of the total variance, with the first factor explaining 23.7%. Therefore, common-method bias did not appear to be a major concern in this study.

### Measures

#### Availability and use of maintenance and development HRM practices

Availability and use of HRM were measured using 20-item scales comprising maintenance and development HRM practices (see Appendix 1). In line with the majority of HRM literature, we measured the perceived availability and use of HRM practices as reflected in the perceptions of the employees (Boselie et al., 2005). Availability was measured by asking respondents whether an HRM practice was available to them at the current employer. Use of HRM was measured using the same items as availability, and measured whether employees had actually made use of an HRM practice.

#### Social support

*Quality of leader-member exchange (LMX)* was measured using the seven-item scale by Janssen and Van Yperen (2004). An example item was: “My employer is helpful with handling problems when they occur in my work.” Answers were provided on a 7-point Likert scale, ranging from “to a very small extent” to “to a very large degree.”

*The quality of coworker exchange (CWX)* was measured using the seven-item scale by Kristensen et al. (2005). An example item was: “People I work with are helpful in getting the job done.” Answers were provided on a 7-point Likert scale, ranging from “to a very small extent” to “to a very large degree.” Cronbach's alpha for the total scale Social support at T1 was 0.97, and at T2.96.

#### Employee outcomes

*Work engagement* was measured using the seventeen-item scale by Schaufeli et al. (2002). An example item was: “At work, I am full of energy.” Answers were provided on 7-point scale, ranging from “never” to “always.”

*Employability* was measured using the fifteen-item scale by Van der Heijde and Van der Heijden (2006). An example item was: “I consider myself competent to engage in in-depth, specialist discussions in my job domain.” Answers were provided on 7-point scale, ranging from “worst capability” to “best capability.”

*Health* was measured using one item: “In general, how would you rate your health?” Answers were provided on a 5-point scale, ranging from “bad” to “excellent.” Cronbach's alpha for the total scale at T1 was 0.94, and at T2.95.

## Statistical analysis

Following Fabrigar et al. (1999), we initially conducted an exploratory principal components analysis using principal components (PCA) on the 20 perceived HRM items at T1 using oblique rotation (Oblimin) for the total sample. The Kaiser-Meyer-Olkin measure verified the sampling adequacy for the analysis, KMO = 0.93 (good according to Field, 2009). Bartlett's test of Spherity $\chi_{\left(190\right)}^{2}$ = 3918.73, *p* < 0.001, indicated that correlations between items were sufficiently large for conducting a PCA. An initial analysis showed that 3 components had Eigenvalues > 1 (Kaiser's criterion), altogether explaining 70.98% of the variance. The scree plot showed two points of inflection at 2 and 3 components. Part-time work turned out to be a component on itself, and was therefore dropped, leading to a two-factorial solution. Table 1 shows the factor loadings after Oblimin rotation of the 19 HRM practices. All items with communalities >0.20 after extraction were included. The item factor loading cut-off point was |0.30| and cross-loading cut-off point was < |0.20|. The two resulting components were labeled as development HRM practices and maintenance HRM practices, respectively. Furthermore, since earlier research (e.g., Gratton and Truss, 2003; Khilji and Wang, 2006; Conway and Monks, 2008; Nishii et al., 2008; Snape and Redman, 2010) has shown that measures of perceived and actual used HRM can differ substantially from intended, implemented HRM, and from each other, we distinguished between perceived and actual used HRM bundles of HRM practices.

**Table 1:** Summary of Exploratory Factor Analysis Results for the HRM Practices (*N* = 228).

**Item**	**Communality**	**Development**	**Maintenance**
4 × 9 working week	**0.61**	0.10	**0.71**
Flexible (beginning and ending) worktime	**0.33**	−0.08	**0.62**
Working from home	**0.60**	0.09	**0.71**
Extra leave or vacation (for “example” free days for leave)	**0.56**	0.03	**0.73**
Dispensation from extra work or overtime	**0.70**	0.00	**0.83**
Long-term absence of work (sabbatical)	**0.65**	−0.06	**0.84**
Variable payment, couples with work prestation	**0.76**	0.03	**0.85**
Flexible working conditions	**0.74**	0.02	**0.85**
Adjusted working conditions	**0.62**	−0.00	**0.79**
Job evaluation (minimum once a year)	**0.55**	0.01	**0.73**
Career guidance	**0.66**	**0.79**	0.04
Permanent development in my function	**0.79**	**0.87**	0.02
Recurrent training or education (minimum once a year)	**0.72**	**0.87**	−0.04
Getting a promotion at work	**0.85**	**0.91**	0.02
Getting a demotion at work	**0.66**	**0.77**	0.08
Horizontal change of function (level does not change)	**0.78**	**0.93**	−0.07
Job enrichment (expansion of function with new tasks)	**0.81**	**0.92**	−0.03
Starting a new career (and retraining) within the organization	**0.78**	**0.87**	−0.02
The possibility to take part in the decision-making within the company	**0.73**	**0.84**	0.03
Eigenvalues		10.56	2.32
% of variance		55.56	12.22

*Communalities > 0.20 in bold, factor loadings > |0.30| in bold, item 1 with cross-loading < |0.20| dropped*.

Structural equation modeling (SEM) techniques utilizing AMOS 21 software (Arbuckle, 2006; Byrne, 2013) were conducted to analyze our panel data. First, we tested a series of measurement models to investigate the operationalization of the two social support factors as an underlying dimension of one overall support factor, and the three employee outcome factors as an underlying dimension of one overall factor for employee outcomes, respectively (see also Luthans et al., 2007). For the first factor, i.e., social support, we compared an uncorrelated, first-order CFA model (wherein all of the 14 social support variables with their respective items were represented as independent constructs) with a second-order CFA model (wherein the items of each scale loaded on the respective underlying factor, i.e., the seven LMX items loaded on one LMX factor, the seven CWX items on one CWX factor, and then the 14 specific social support variables loaded on one overall social support factor). We conducted this analysis twice, that is, separately for the two different measurement models. The same procedure was performed for the employee outcomes. Results supported the representation of LMX and CWX in one overall social support factor, since the second-order model showed an acceptable and significantly better fit in comparison with the first-order model [for T1: $\Delta \chi_{\left(13\right)}^{2}$ = 224.76, *p* < 0.001; for T2: $\Delta \chi_{\left(13\right)}^{2}$ = 189.14, *p* < 0.001]. There was also evidence for the representation of work engagement, employability, and health in one general employee work outcomes factor [for T1: $\Delta \chi_{\left(70\right)}^{2}$ = 837.05, *p* < 0.001; for T2: $\Delta \chi_{\left(96\right)}^{2}$ = 936.11, *p* < 0.001]. The output of these analyses are available from the first author upon request.

Given our relative small sample size, we decided to reduce the complexity of our hypothesized SEM models (i.e., number of free estimated parameters) by using manifest variables (see Jöreskog and Sörbom, 1993 for more specific information). In order to use the scores for the “social support,” and “employee outcomes” manifest variables, we calculated their weighted factor scores. Specifically, we used Varimax rotation and conducted second-order principal axis factoring (PAF) analysis on the two variables of social support, and the three employee outcomes variables at both measurement times. PAF analyses resulted in one social support factor (84% of explained variance at T1 and 80% at T2), and one employee outcome factor (59% of explained variance at T1 and 63% at T2). Thus, the manifest variable for “social support” represented the factor score of the two social support scales, and the manifest variable “employee outcome” represented the factor score of the three employee scales. PAF analyses of each HRM bundle separately resulted in four factors (56% of the variance was explained at T1, and 55% at T2).

To test the research hypotheses a number of competing models were fitted to the data consecutively by means of a cross-lagged structural (Zapf et al., 1996) equation model approach. First, a *stability model* (M1) without cross-lagged structural paths, but including the correlations between the constructs for each possible pair of measurement waves (see Pitts et al., 1996; Salanova et al., 2006) and synchronous correlations was specified. This model was compared with the other models representing each of the hypotheses. Compared to the stability model, the second model (M2) included additional structural paths from T1 HRM bundles to T2 employee outcomes. The third model (M3) was identical to the stability model as well but included all paths from the previous models, as well as paths from T1 HRM bundles to T2 social support, and from T2 social support to T2 employee outcomes.

The fit of the distinguished models to the data was assessed with the chi-square (χ^2^) statistic, the Goodness of Fit Index (GFI), and the Root Mean Square error of Approximation (RMSEA). In addition, the Comparative Fit Index (CFI), the Incremental Fit Index (IFI), and the Tucker-Lewis Index (TLI) were used as these are less sensitive to the data. For all of these statistics, values of 0.90 are acceptable and 0.95 or higher are indicative of good fit (Hu and Bentler, 1999), except for the RMSEA for which values of 0.05 indicate good fit and values up to 0.08 represent reasonable errors of approximation (Browne and Cudeck, 1993).

## Results

### Descriptive analysis

Cronbach's alpha coefficients and bi-variate correlations (including test-retest correlations) of the study variables were computed, before further model testing (see Table 2 for all specific outcomes). All variables had test-retest reliabilities of 0.41 on average. The highest test-retest reliabilities resulted for social support and employee outcomes. This means that social support and employee outcomes are relatively stable experiences. In addition, all significant correlations were in the expected direction. Cronbach's alpha for all constructs ranged from 0.65 to 0.97, and thus provided satisfactory internal consistency at both measurement times.

**Table 2:** Means, Standard Deviations, Cronbach's Alphas (in Brackets on the Diagonal) and Inter-correlations among Study Variables (*N* = 228).

	**Variable**	***M***	***SD***	**1**	**2**	**3**	**4**	**5**	**6**	**7**	**8**	**9**	**10**	**11**	**12**
**TIME 1**															
1	Perceived maintenance	0.24	0.33	(0.93)											
2	Perceived development	0.23	0.36	0.64^**^	(0.96)										
3	Used maintenance	0.12	0.19	−0.05	0.04	(0.80)									
4	Used development	0.10	0.22	0.08	−0.07	0.56^**^	(0.91)								
5	Social support	4.47	1.33	−0.08	0.03	0.09	0.08	(0.97)							
6	Employee outcomes	4.51	0.59	−0.10	−0.03	0.10	0.12	0.36^**^	(0.94)						
**Time 2**															
7	Perceived maintenance	0.14	0.23	0.19^**^	0.33^**^	−0.02	−0.05	0.16^*^	−0.03	(0.86)					
8	Perceived development	0.17	0.29	0.22^**^	0.39^**^	0.06	0.05	0.02	0.05	0.62^**^	(0.92)				
9	Used maintenance	0.11	0.14	−0.01	0.06	0.38^**^	0.25^**^	0.04	0.13^**^	0.04	0.24^**^	(0.65)			
10	Used development	0.07	0.16	−0.07	−0.02	0.07	0.18^**^	−0.00	0.14^*^	0.06	0.07	0.38^**^	(0.80)		
11	Social support	5.01	1.16	0.06	0.07	0.06	0.10	0.55^**^	0.39^**^	0.08	0.07	0.19^**^	0.00	(0.96)	
12	Employee outcomes	4.48	0.60	−0.04	0.02	0.06	0.07	0.33^**^	0.77^**^	0.04	0.07	0.12	0.09	0.53^**^	(0.95)

* *p < 0.05*;

** *p < 0.01*.

### Hypotheses testing

To explore the effects of HRM bundles on employee outcomes (*H1*), and the hypothesized mediation of social support (*H2*), a longitudinal two-wave path analysis for the total group was performed. Our conceptual model yielded fit statistics that indicated a good model (M3) fit.

Table 3 shows the fit indices of the competing models. The stability model (M1) and M2 appeared to have a bad fit to the data. M3 was the only model with a very good fit to the data, with all fit indices being higher than 0.90 and with the RMSEA being lower than 0.05. In addition, the ratio between the chi-square statistic and the number of degrees of freedom was relatively low. As regards the model comparisons, most importantly, the χ^2^ difference tests showed that both M2 was not superior to M1 [$\chi_{\left(4\right)}^{2}$ = 1.45, *p* = 0.84].This outcome indicates that the inclusion of paths either from perceived or used, and either maintenance or development HRM bundles to employee outcomes, does not improve model fit. Nevertheless, Table 3 shows that M3 fitted significantly better to the data than M1 and M2 [$\chi_{\left(10\right)}^{2}$ = 461.02, *p* < 0.001, and $\chi_{\left(6\right)}^{2}$ = 459.57, *p* < 0.001, respectively]. This indicates that the theoretical model including cross-lagged relationships between HRM bundles, social support, and employee outcomes fitted best the empirical data.

**Table 3:** Goodness-of-Fit Indices for the Alternative Models (*N* = 228).

**Model**	**χ^2^**	***df***	***P***	**AGFI**	**GFI**	**RMSEA**	**CFI**	**NFI**	**TLI**	**IFI**
M1. Stability model	532.67	60	0.000	0.69	0.76	0.19	0.44	0.41	0.38	0.44
M2. HRMT1 → EOT2	531.22	56	0.000	0.67	0.76	0.19	0.43	0.41	0.33	0.44
M3. HRMT1 → SST2/EOT2, SST2 → EOT2	71.65	50	0.024	0.92	0.95	0.044	0.97	0.92	0.97	0.98
Null model	903.05	66	–	0.55	0.62	0.24	–	–	–	–

*HRM, human resource management; SS, social support; EO, employee outcomes; T1, Time 1; T2, Time 2; AGFI, adjusted goodness-of-fit index; GFI, goodness-of-fit index; RMSEA, root mean square error of approximation; CFI, comparative fit index; NFI, normed fit index; TLI, Tucker-Lewis index; IFI, incremental fit index*.

We will now go into the specific structural relationships using the best fitting model. Hypothesis 1 asserted that perceived availability and used maintenance and development HRM bundles at Time 1 are positively related to employee outcomes at Time 2 among a sample of bridge workers. The model that included these causal relationships, i.e., M2, did not result in any statistically significant lagged and positive effects of T1 HRM bundles on T2 employee outcomes. The standardized betas varied from −0.02 to 0.03, *ps* > 0.05). Therefore, Hypothesis 1 was not supported.

**Figure 2:**
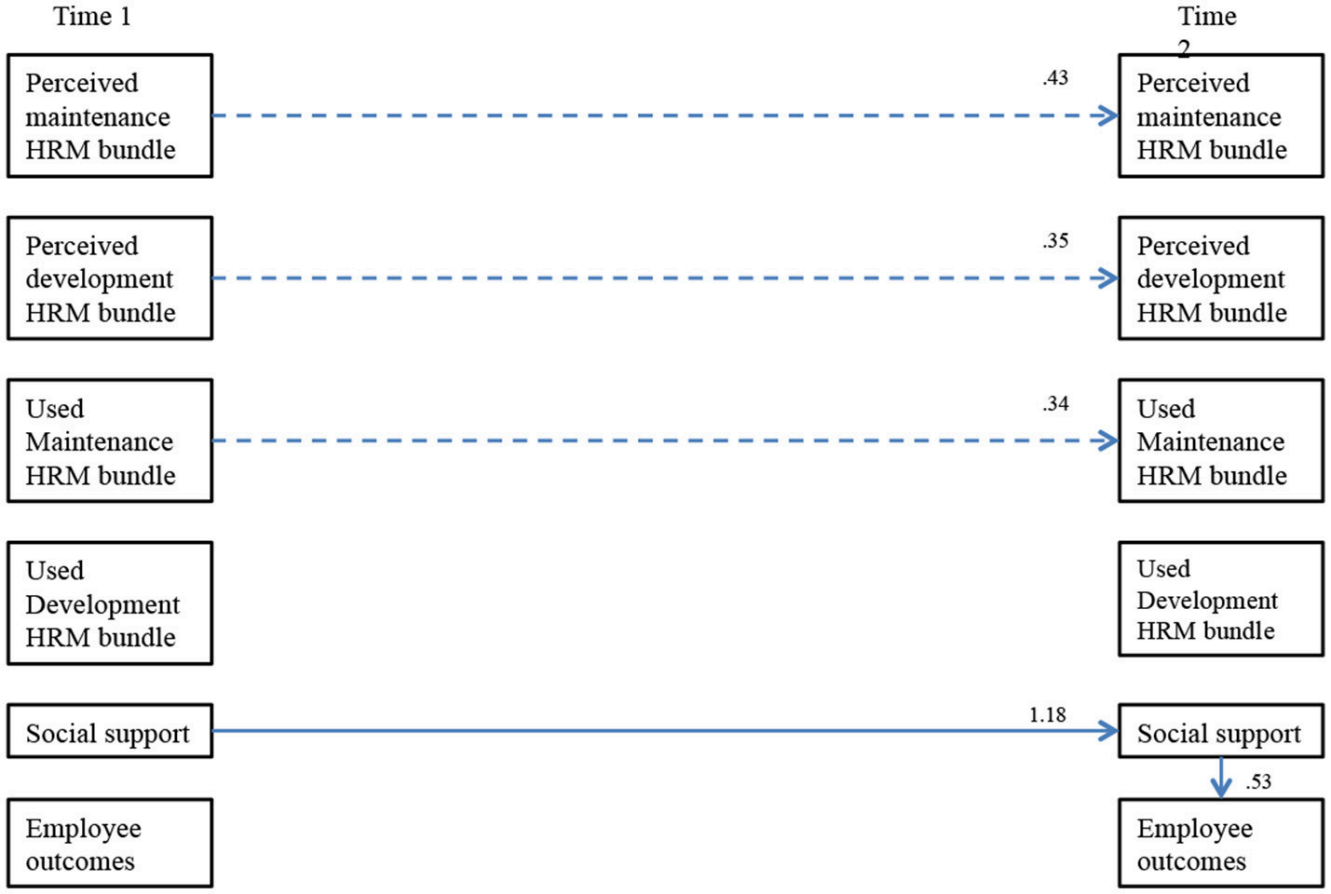
The hypothesized model, *N* = 228. Non-significant correlations are omitted for the sake of clarity. Significant standardized regression weights are depicted above the arrows indicating the structural relationships. Hypothesized regression weights are depicted with solid lines, and non-hypothesized regression weights with dotted lines.

Hypotheses 2 stated that T2 social support mediates the relationship between Time 1 perceived and used HRM and Time 2 employee outcomes in a sample of bridge workers. Following James et al. (2006) and Schneider et al. (2005), we tested both a full and partial mediation model. There appeared no evidence for partially mediated effects. Figure 2 presents the results for the full mediation model. The outcomes of the fully mediated Model 3 (M3) including causal paths from T1 HRM bundles and T2 social support on T2 employee outcomes, resulted in several unexpected cross-lagged relationships. Specifically, none of the T1 HRM bundles appeared to have a significant impact on T2 employee outcomes, nor on T2 social support. Nevertheless, T1 social support positively impacted social support at T2, which in turn, positively influenced employee outcomes at T2. Furthermore, an in-depth analysis showed nearly the same regression weights for the relationships between LMX and CWX at T1, on the one hand, and LMX and CWX at T2, on the other (β = 0.47 and β = 0.48, respectively), and between LMX and CWX T2 to employee outcomes T2 (β = 0.27 and β = 0.29, respectively). The statistically significant paths in M3 overlapped with those of M1 and M2, and are displayed in Figure 2. For sake of clarity, the non-significant paths were not depicted. In addition, dotted lines represent significant results that were not captured in the hypotheses. We found significant relations between three HRM bundles at T1 and at T2. With these outcomes, Hypothesis 2 was partly supported, in the sense that the positive effects on employee outcomes T2 were solely caused by social support T2, and not by perceived and used T1 HRM bundles.

## Discussion

The main purpose of the present study was to examine the longitudinal relationships between HRM bundles of practices (perceived versus used, and maintenance versus development), social support, and employee work outcomes among a unique panel of 65+ bridge workers. Based on the social exchange theory (Gouldner, 1960; Blau, 1964) and the JD-R framework (Demerouti et al., 2001; Bakker et al., 2003), it was hypothesized that HRM bundles would influence employee outcomes of bridge employees, and that this relationship would be mediated by social support over time.

On the one hand, our data strongly support the assumption about the impact of high levels of job resources, such as social support (see Gouldner, 1960; Blau, 1964; Demerouti et al., 2001; Bakker et al., 2003, 2007; Cropanzano and Mitchell, 2005) on high levels of employee outcomes, such as more work engagement, employability, and perceived health. Specifically, a sustained relationship from the employee with the supervisor and colleagues has significant impact on employee outcomes. Therefore, drawing on both social exchange theory (Gouldner, 1960; Blau, 1964) and the JD-R framework (Demerouti et al., 2001; Bakker et al., 2005), we identified two resources that are critical for positive employee outcomes, i.e., LMX and CWX. Hence, this study shows the importance of the unique relationship of a supervisor, and also of colleagues to each employee, that can significantly influence their “bridge subordinates” or “bridge colleagues” attitudes and behavior (Liden et al., 1993; Graen and Uhl-Bien, 1995; Gerstner and Day, 1997; Ilies et al., 2007). This finding implies that the retirement decisions might be influenced by, as individuals age, prioritization of present emotionally meaningful -rather than knowledge-related- goals, due to limited future time perspective (Lang and Carstensen, 2002). As stated earlier, according to the Socioemotional Selectivity Theory (SST; Carstensen et al., 1999; Carstensen, 2006), grounded in the uniquely human ability to monitor time, chronological age is inversely associated with actual and perceived time left in life. Hence, in comparison to younger people, older people in particular enjoy relatively stable and positive emotional experiences in daily life, and prioritize meaningful activities over activities that are related to individual achievement and exploration. This supports our finding of the importance for bridge workers of positive relationships with supervisors and colleagues. Related to this statement, 65+ workers might emphasize personal constructivism over social constructionism, in which human development is seen as driven by adaptation to a social environment with the goal of person-environment integration, rather than by maturation of inner structures (Savickas, 2012). Thus, after a more than 30 years period, occupation and organizations often have changed dramatically, and may result in a declined person-environment fit (Feldman and Beehr, 2011). Older workers may find themselves in jobs they no longer find rewarding, or perceive declines in cognitive processing or physical abilities that occur with aging (Kanfer and Ackerman, 2004). Resources, such as good relationships with supervisors and colleagues seem to be of increased importance.

On the other hand, for bridge workers the organizational adaptation demonstrated no relation to the more distant HRM bundles. Contradictory to our assumptions and notwithstanding the “healthy worker effect,” our four constructed HRM bundles that built and support LMX and CWX (see also Wright et al., 2001) did not evoke employee outcomes. Bridge workers as the healthy workers in optima forma, were expected to be more prone to positive work outcomes, since the ill and chronically disabled work population are ordinary excluded form employment (Last, 1995). However, economically active 65+ workers appeared to not reciprocate positively to the HRM bundles provided by the organizations. We may argue that 65+ workers have a fundamentally different relationship with their organization than younger workers (see also Veth et al., 2017). Possibly, their intent for a long-term investment has obtained another connotation, since—as stated before—their future time perspective has changed (Lang and Carstensen, 2002). As older people perceived their future time as more limited than younger people, emotionally meaningful goals get prioritized, whereas younger people with a more open-ended future time perspective prioritize more knowledge-related goals. Therefore, an HRM bundle including regular training (part of development bundle) may not improve the situation of the economically active 65+ workers. In addition, an HRM practice such as extra leave (part of maintenance HRM bundle) appeared not to improve the employee outcomes either. This might be related to the fact that bridge workers continue working on a part-time basis (in our study, on average, 14.25 h a week). Thus, strains bridge workers had to cope with during their working life before retirement, seem to be decreased. It seems that a demarcation, in this case between the age of 65, separating these employees from their younger counterparts makes sense (see also Lang and Carstensen, 2002). Indeed, for instance, Veth et al. (2015) found that 55+ workers evaluated development HRM as successful as maintenance, and indicated to have relatively higher needs for the first category. This differentiating finding might enlarge Super's stage theory (1951, 1957, 1990), going beyond the final stage of career. As such, the final stage is characterized by disengagement from and decline in work activities. However, as the future time perspective shortens, a distinction emerges between individuals who seek out to bridge employment and those who retire fully. In line with the role theory, in case workers are dissatisfied with their career, they may seek to escape the undesirable situation by taking a role exit (Wang, 2007). The role exit may either be full retirement or a switch to different occupational field for bridge employment. In contrast, to the extent employees have highly invested in and are satisfied with their current work role, they may seek a way to remain in a job as similar as possible to their position before retirement. Wang et al. (2008) emphasized this distinction, stating that all bridge jobs are not alike: career bridge employment (i.e., jobs similar to positions before retirement) and non-career employment (i.e., entail working areas that are not directly related to preretirement employment). Anyway, whether older workers are motivated by approach or avoidance (see also Elliot and Harackiewicz, 1996; Hamamura et al., 2009) to choose to engage in bridge employment, bridge employment allows older workers to keep one foot in work and one foot in retirement in which they do not rely on HRM.

A practical implication is that managers are urged to optimize their relations with their 65+ employees, and facilitate optimized relations with colleagues. More specifically, these relations need to have a sustained character, and cannot be built overnight. Beliefs about respect, trust, and loyalty are as important for a fruitful relationship with both the supervisor and the colleagues (Graen and Uhl-Bien, 1995; Murphy and Ensher, 1999; Schriesheim et al., 1999; Ilies et al., 2007; Nishii and Mayer, 2009). However, both maintenance and development HRM bundles do not enhance employee outcomes of economically active 65+ workers. HRM bundles might thus be perceived as hygiene factors, though a decrease of employee outcomes might result from the absence of HRM. Organizations might provide HRM bundles specifically targeted at bridge workers in a sense that these bundles should be more “close” to the work place. Whereas a training for the future is not appropriate, on the job learning for instance, might fit the bridge worker.

This empirical work has some limitations. First, all data were obtained by using surveys and by using only self-reports for the predictor and outcomes variables, herewith opening up the possibility of response set consistencies. Common-method bias is a possible threat to the validity of conclusions in case of only using questionnaires for data collection in empirical scholarly work. However, as we reported in the Sample and Procedure section this was not a major concern in the current study. In future research we nevertheless recommend to supplement the current survey approach with other data collection methods, such as multi-source ratings although these may be prone to stereotyping or halo-effects (Kerlinger Fred and Lee Howard, 2000). In particular, it would be interesting to incorporate additional objective ratings in future research.

Despite our longitudinal design, allowing a time interval among hypothesized predictors and outcomes, we are not able to draw definite conclusions about causality. For instance, an effect of a particular Time 1 predictor on a specific Time 2 outcome variable might also be due to an unmeasured third variable. In our study, however, the importance for bridge workers of social support, rather than HRM bundles, is not contradicted.

This study is based on a fairly small number of participants, which limits the generalizability of our results. However, the participants were spread across several sectors and included various jobs and tasks on different levels. This improved the heterogeneity of our sample. Furthermore, the current study was focused on bridge workers, whereas it might be interesting to compare the outcomes among different age groups as well. Next, although the advantage of this study is its longitudinal character, the time-interval was based on a one-year time lag. Dormann and Zapf (2002) stated that in many situations this time lag is chosen pragmatically, whereas a two-years time lag seems to be more adequate. Although the current study has produced noteworthy insights, we encourage future scholarly work to use a time lag of 2 years in order to test the robustness of the relationships between work characteristics and well-being.

Notwithstanding these limitations, our study concerned the first longitudinal study to date to address the longitudinal effects among HRM, social support, and employee (65+) outcomes. The results have shown that to sustain bridge workers at work, employers should mainly focus on creating high-quality relationships with supervisors and colleagues, instead of providing HRM bundles for bridge workers.

## Ethics statement

We hereby confirm that this manuscript has not already been published and that this manuscript is not being considered for publication elsewhere. In addition, ethics approval and written consent for the study are in accordance with the Dutch Association of Psychologists, the professional body of psychologists in the Netherlands. Also, institutional guidelines of our employers have been carefully taken into account according the NIP's code of ethics. Given the character of the data, no further written informed consent was needed.

## Author contributions

KV and BV: theoretical framework, method, results, discussion, and overview; HK: method, results; AD: theoretical setup, data; BE: discussion and overview.

### Conflict of interest statement

The authors declare that the research was conducted in the absence of any commercial or financial relationships that could be construed as a potential conflict of interest.

## Appendix 1

1. Part-time work
2. 4 × 9 working week
3. Flexible (beginning and ending) working time
4. Working from home
5. Extra leave or vacation (for example: free days for leave)
6. Dispensation from extra work or overtime
7. Long-term absence of work (sabbatical)
8. Variable payment, coupled with work performance
9. Flexible working conditions
10. Adjusted working conditions
11. Job evaluation (minimum of once a year)
12. Career guidance
13. Permanent development in my function
14. Recurrent training or education (minimum: once a year)
15. Getting a promotion at work
16. Getting a demotion at work
17. Horizontal change of function (level does not change)
18. Job enrichment (expansion of function with new tasks)
19. Starting a new career (and retraining) within the organization
20. The possibility to take part in the decision-making within the company
